# Ontogenetic Variation in Biological Activities of Venoms from Hybrids between *Bothrops erythromelas* and *Bothrops neuwiedi* Snakes

**DOI:** 10.1371/journal.pone.0145516

**Published:** 2015-12-29

**Authors:** Marcelo Larami Santoro, Thaís do Carmo, Bruna Heloísa Lopes Cunha, André Fonseca Alves, André Zelanis, Solange Maria de Toledo Serrano, Kathleen Fernandes Grego, Savio Stefanini Sant’Anna, Katia Cristina Barbaro, Wilson Fernandes

**Affiliations:** 1 Laboratório de Fisiopatologia, Instituto Butantan, São Paulo-SP, Brazil; 2 Laboratório Especial de Toxinologia Aplicada and Center of Toxins, Immune-Response and Cell Signaling, Instituto Butantan, São Paulo-SP, Brazil; 3 Laboratório de Herpetologia, Instituto Butantan, São Paulo-SP, Brazil; 4 Laboratório de Imunopatologia, Instituto Butantan, São Paulo-SP, Brazil; Universidad de Costa Rica, COSTA RICA

## Abstract

Lance-headed snakes are found in Central and South America, and they account for most snakebites in Brazil. The phylogeny of South American pitvipers has been reviewed, and the presence of natural and non-natural hybrids between different species of *Bothrops* snakes demonstrates that reproductive isolation of several species is still incomplete. The present study aimed to analyze the biological features, particularly the thrombin-like activity, of venoms from hybrids born in captivity, from the mating of a female *Bothrops erythromelas* and a male *Bothrops neuwiedi*, two species whose venoms are known to display ontogenetic variation. Proteolytic activity on azocoll and amidolytic activity on N-benzoyl-DL-arginine-*p*-nitroanilide hydrochloride (BAPNA) were lowest when hybrids were 3 months old, and increased over body growth, reaching values similar to those of the father when hybrids were 12 months old. The clotting activity on plasma diminished as hybrids grew; venoms from 3- and 6-months old hybrids showed low clotting activity on fibrinogen (i.e., thrombin-like activity), like the mother venom, and such activity was detected only when hybrids were older than 1 year of age. Altogether, these results point out that venom features in hybrid snakes are genetically controlled during the ontogenetic development. Despite the presence of the thrombin-like enzyme gene(s) in hybrid snakes, they are silenced during the first six months of life.

## Introduction

Delimitation and definition of the category “species” is controversial. Among other problems, this occurs because in the past morphological characters were primarily used as a major criterion to distinguish species. In several occasions, species considered diverse may show similar morphological features, and vice versa, making species differentiation laborious. In addition, there is no universally accepted consensus for what a species is, and many definitions have been proposed [1,2]. Among those definitions, some deserve special attention, such as the biological species concept proposed by Mayr [2], which takes into account the reproductive isolation among groups of organisms. Other concepts, like the phenetical, phylogenetical and ecological, emphasize various biological properties other than reproductive isolation [3,4]. In addition, there are several evolutionary processes that make species delimitation difficult; perhaps the most important among them is “mosaic evolution”, whereby certain characters evolve more rapidly than others, resulting in a disharmony within the assembly of several characters. Thus, reproductive isolation does not always parallel morphological differentiation [2].

*Bothrops* (*sensu lato*) snakes are widely distributed in South America, and in 2013–2014 they inflicted 87% of snakebites occurring in Brazil [5]. According to the taxonomic classification found elsewhere [6], there are 23 species of *Bothrops* snakes in Brazil. Despite their wide distribution, however, few pieces of information are available for their natural history and phylogeny. In fact, there is no unanimity among the phylogeneticists or evolutionists about the taxonomical classification of the genus *Bothrops* (*sensu lato*), and various phylogenetic classifications have been reported [7–13].

Gene exchange by hybridization and introgressive hybridization are important mechanisms to engender organism diversity [14]. Hybridization was defined by R. G. Harrison as “the interbreeding of individuals from two populations, or groups of populations, which are distinguishable on the basis of one or more heritable characters”, while R. H. Rieseberg and J. F. Wendel defined introgressive hybridization as “the permanent incorporation of genes from one set of differentiated populations into another, i.e. the incorporation of alien genes into a new, reproductively integrated population system” (apud [14]). Hybridization, i.e., the interbreeding between two closely related taxa, has been acknowledged as a mechanism which contributes to speciation [14,15]. One intriguing question about taxonomical classification in *Bothrops* (*lato senso*) is the existence of reports on natural and non-natural hybrids, e.g., between *Bothrops neuwiedi* and *Bothrops alternatus*, *B*. *alternatus* and *Bothrops cotiara* [16], and *Bothrops jararaca* and *Bothrops jararacussu* [17], and non-natural hybrids between *B*. *jararaca* and *B*. *neuwiedi* [18]. Although snake venoms are recognized as complex mixtures of toxins subject to variation at all taxonomical levels, as well as to sexual dimorphism, seasonal variation, ontogenetic development, habitat, geographical origin and diet [19], only three studies have investigated features of venomous hybrid snakes [20–22]. Mostly, hybrid snakes have been characterized mainly by their morphological features and genotype [23–29].

**Fig 1:**
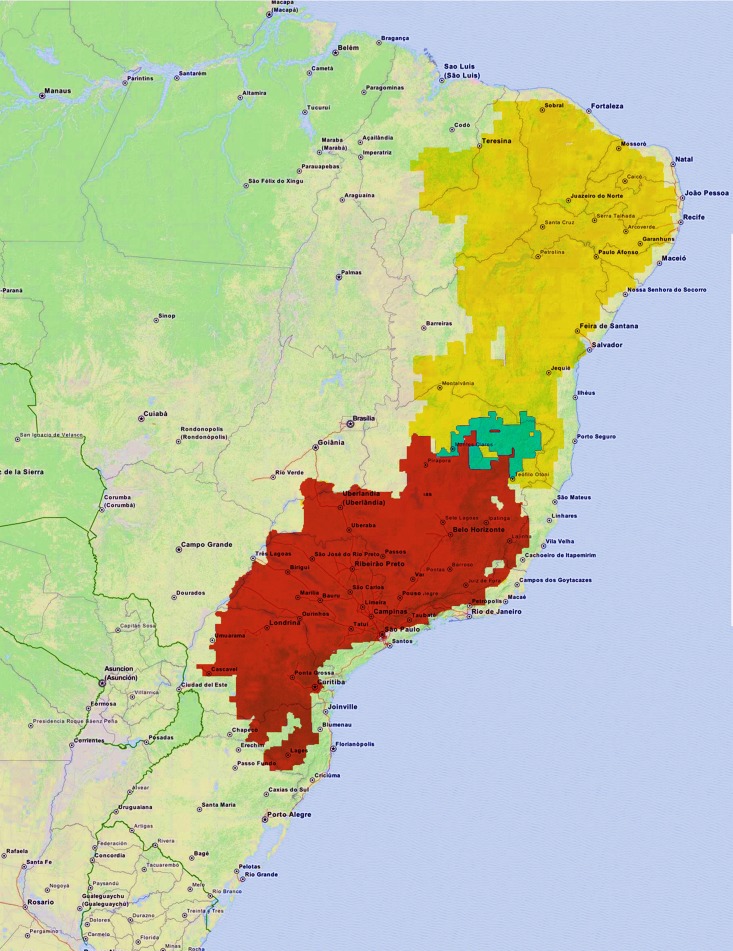
Illustrative geographical distribution of *Bothrops erythromelas* (yellow area) and *Bothrops neuwiedi* (red area) in Brazil. Areas of sympatria are shown in cyan. Distribution data were based on [39]. Brazil map was obtained from OpenStreetMap contributors (http://www.openstreetmap.org/).

*Bothrops erythromelas* and *B*. *neuwiedi* have been grouped in *Bothrops neuwiedi* group ([30] and references therein), a monophyletic group, but *B*. *erythromelas* has been shown to be the sister group to the remaining *B*. *neuwiedi* group [30]. The snake *B*. *erythromelas* is terrestrial, 40–50 cm long, and inhabits xeric and semiarid thornforest, dry tropical deciduous forest and open rocky areas mainly in the northeastern states of Brazil [6] (Fig 1). On the other hand, *B*. *neuwiedi* is terrestrial and is ca. 60–70 cm long; it inhabits tropical and semitropical deciduous forest, temperate forest, and sand ridges along the coast (*restingas*) on Atlantic forest in east and southeast states of Brazil [6]. *B*. *erythromelas* may be sympatric with *B*. *neuwiedi*, and indeed pieces of evidence suggest introgressive hybridization in *B*. *erythromelas* [30]. The venoms from both species show the characteristic triad of inflammatory, hemorrhagic and coagulating activity of *Bothrops* venoms, and bites inflicted by these species cause inflammatory reaction at the site of the bite, and hemostatic disturbances [31,32]. *B*. *erythromelas* venom is particular among other *Bothrops* species due to its lack of thrombin-like activity, which convert directly fibrinogen into des-A fibrin, and to exhibit high amounts of factor II and factor X activators [33,34]. However, variation in the coagulation activity of *B*. *erythromelas* on plasma and fibrinogen has been reported [35–37]. In addition, *B*. *erythromelas* displays ontogenetic shift, but in both newborn and adult venom samples the amidolytic activity on Nα-benzoyl-DL-arginine-*p*-nitroanilide hydrochloride (BAPNA) [38] and thrombin-like activity are negligible. Newborn *B*. *neuwiedi* venom also shows ontogenetic shift, illustrated by the fact that the venom from newborn specimens exhibits higher prothrombin and factor X activating, similar amidolytic and thrombin-like activities, and lower fibrinolytic activity in comparison with their mother venoms [38].

We characterized herein venoms from a litter from hybrid snakes, born in captivity, from the mating of a female *B*. *erythromelas* and a male *B*. *neuwiedi*. These snakes inhabit two diverse geographical regions in Brazil, but they coexist within hybrid zones. Both individuals were temporarily maintained together in the Laboratory of Herpetology (Butantan Institute, São Paulo, Brazil), and then mated, resulting in an offspring of 15 individuals. Since most reports in literature about hybridism in snakes mainly focused on their external morphological characters, we took advantage of this unique opportunity to study one major question: are venoms from hybrid snakes a simple combination of characters from both genitors, or do they vary over their ontogenetic development like their parent species venoms? Moreover, once newborn and adult *B*. *erythromelas* venom has been reported to be devoid of thrombin-like activity, we engaged in studying whether hybrid venoms displayed this trait in some stage of their ontogenetic development. We observed that hybrid venoms show ontogenetic variation, and that thrombin-like activity is present only when hybrids are beginning to become adults.

## Materials and Methods

### Animals and venom samples

All procedures involving the use of animals were approved by the Ethical Committee for the Use of Animals of Institute Butantan (certificates 488/08 and 806/11).

**Fig 2:**
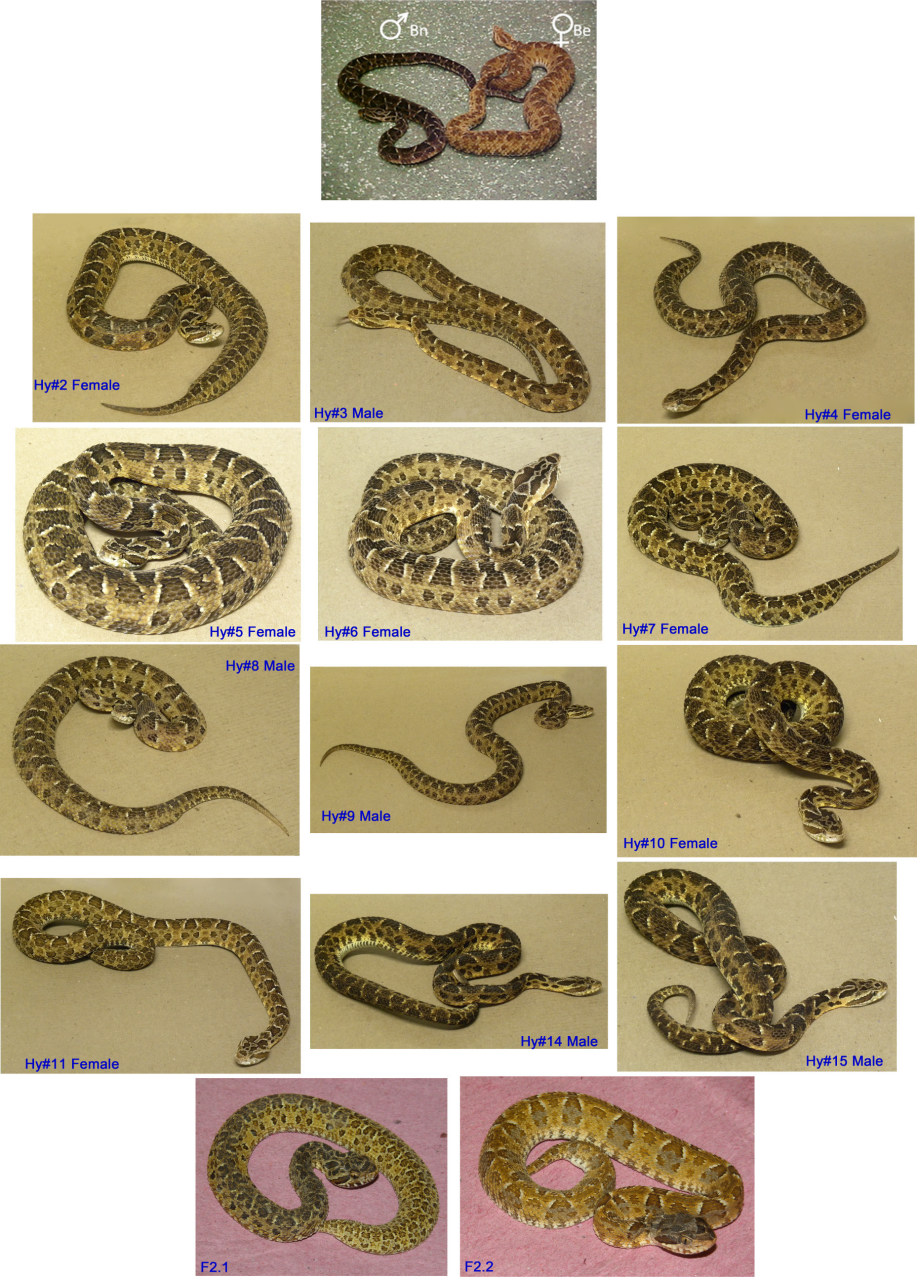
Photographs of hybrid snakes. At the top of the figure, the *B*. *erythromelas* (Be, ♀) and the *B*. *neuwiedi* (Bn, ♂) that mated and originated F1 hybrids (Hy) (photographed at 36 months old). At the bottom of the figure is shown two 2-month-old individuals originated from the mating between Hy# 11 and Hy# 03.

On June 8, 2006, one female specimen of *Bothrops erythromelas*–born in captivity in 2003 from a mother captured in Brumado (Bahia State, Brazil)–was left to mate with two specimens of male *Bothrops neuwiedi* specimens from Baependi (Minas Gerais State, Brazil), which have been kept in captivity since 2002. Such animals were maintained in the Laboratory of Herpetology, Butantan Institute. Agonistic behavior was observed between males, and the dominant individual mated with the female. On February 22, 2007, an offspring (F1) of 15 individuals (8 females, 6 males and 1 stillborn) was born in captivity [40] (Fig 2). Each newborn snake was identified by a number and maintained in individual cages at room temperature (22–28°C). Three hybrids died, one on day one (Hy# 01), one on month seven (Hy #13) and one on month 22 (Hy# 12) after the birth. Snakes were fed every 15 days with neonate mice until they were 12 months old, and thereafter they fed on adult mice once per month at the same frequency, and had free access to water.

Venom samples have been extracted from May 22, 2007, in 3-month intervals. At the moment of extraction, the weight, total length and snout-vent length (SVL) of each animal were obtained. Extracted venoms were centrifuged at 10000 *g* for 5 min, and the supernatant was lyophilized, and individually stored at –80°C. Since hundreds of venom samples were available to analyses, the samples from 6 hybrids were randomly chosen (males Hy# 03, Hy# 08 and Hy# 15, and females Hy# 06, Hy# 10 and Hy# 11), and their venoms were analyzed when they were 3, 6, 12, 24 and 36 months old. The biological mother of hybrids died of an unknown cause in October 2008, when hybrids were ca. 19 months old, so that her venom was not analyzed when hybrids were 24 and 36 months old.

For experiments of one- and two-dimensional gel electrophoresis, and Western blotting, venom pools were also produced by mixing identical amounts of individual venom samples. Similarly, venom pools were obtained from the mother (from 6 extractions), the biological father (from 10 extractions), young hybrids (age: 3 and 6 months old; samples from 2 extractions from 14 hybrids), and adult hybrids (age: from 2 years old onwards; samples from 5 extractions from 11–12 hybrids).

### Coagulant activity

Initially, the coagulant activity of venom samples was evaluated by determining the minimum coagulant dose (MCD) [41] on bovine plasma (total coagulant activity) or 2 mg/mL bovine fibrinogen solution (thrombin-like activity), as described elsewhere [42]. In brief, 100-μL aliquots of citrated bovine plasma or bovine fibrinogen solution (2 mg/mL) were maintained at 37°C for 2 min, and thereafter aliquots (25 μL) of two-fold serially diluted venom samples were added, and the clotting time recorded. Clotting times were measured in triplicate on the mechanical coagulometer Start4 (Diagnostica Stago, France) at 37°C. However, several venom samples showed weak coagulant activity, not allowing the determination of MCD, and thus an index of coagulant activity was devised to obtain a value that could be compared. This index was calculated by obtaining the inverse of clotting time elicited by 1 mg/mL venom solution, and by multiplying it by 100.

### Amidolytic activity

To assay the activity of serine proteinases (SVSP) in venom samples, BAPNA (Sigma, USA) was used as the substrate to evaluate the amidolytic activity in venom samples [42]. Briefly, aliquots (30 μL) of two-fold serially diluted venom samples were incubated with 140 μL of 1 mM substrate solution, both in Tyrode buffer (137 mM NaCl, 2.7 mM KCl, 3.0 mM NaH_2_PO_4_, 10 mM HEPES, 5.6 mM dextrose, 1 mM MgCl_2_, 2 mM CaCl_2_, pH 7.4), for 30 min at 37°C. Reaction was halted by adding 50 μL of 30% (v/v) acetic acid, and the absorbance was read at 405 nm in a SpectraMax 190 microplate reader. The coefficient of extinction of *p*-nitroaniline was considered as $\varepsilon_{405\;nm}^{1\;mM}$= 9.65/cm, and specific activity was expressed in nmol *p*-nitroaniline/min/mg lyophilized venom.

### Collagenolytic activity

Azocoll (Sigma, USA) was used to evaluate the collagenolytic activity of venom samples [42]. Briefly, 50 μL of 5 mg/mL azocoll solution was incubated with 200 μL venom solutions (125 and 62.5 μg/mL), both in Tyrode buffer, at 37°C for 1 h, under homogenization. The reaction was interrupted by placing samples on ice, and after centrifugation at 5000 *g* for 3 min, the absorbance of the supernatant was read at 540 nm. One unit of enzymatic activity was defined as the amount of venom that causes an increase of 0.001 units of absorbance per min at 540 nm. Specific activity was expressed as U/min/mg lyophilized venom.

### One-dimensional gel electrophoresis

Non-reduced samples of venoms (20 μg/lane) were electrophoresed in 12% SDS-polyacrylamide gels as described previously [43].

### Two-dimensional gel electrophoresis, in-gel protein digestion and mass spectrometry (MS) identification

Analyses of two-dimensional electrophoresis were carried out with both individual and pooled venom samples. For individual analyses, venom samples from 3-month old (Hy# 3, 6, 8, 10, 11, 15) and 2-year old (Hy# 3, 6, 8, 10, 11, 15) hybrids were used, as well as from the biological father (venom was extracted when hybrids were 3, 6, 12 and 24 months old) and mother (venom was extracted when hybrids were 3, 6, and 12 months old). The protocol used for two-dimensional electrophoresis was based on a previous report for *B*. *jararaca* venom [42]. Protein content in venom samples (1.0 mg/mL) was determined by BCA assay on microplates [44], using bovine serum albumin as standard. In brief, venom samples (640 μg/mL) were dissolved in rehydration solution, and aliquots (125 μL, 80 μg protein) were used to hydrate precast strips for isoelectric focusing (IEF) (7 cm, linear pH 3–10, GE Healthcare, USA) during 18 h at room temperature. The first dimension IEF was performed in an Ettan IPGphor 3 instrument (GE Healthcare, USA), using the following protocol: 100V-300 Vh, 300V-150 Vh (gradient), 1000V-300 Vh (gradient), 5000V-4000 Vh (gradient) and 5000V-3000 Vh. After IEF, strips were reduced and alkylated, and directly applied to 14% SDS-PAGE gels in a SE260 cell (GE Healthcare, USA), which was connected to a Multitemp III cooling bath (GE Healthcare, USA) set at 10°C. Gels were run at 30 mA/gel until the dye front reached the gel bottom. Thereafter gels were silver stained [45] and scanned on a Epson Imagescanner III, with resolution of 600 dpi. For analyses, each venom sample was run in duplicate, and then images were analyzed with the software ImageMaster 2D 7.0 (GE Healthcare, USA) for determination of relative molecular mass and p*I* of spots. In addition, in order to compare spots from hybrids and their parents, gel images were colorized in Photoshop CS4 Extended and overlapped using the Linear Burn option in the Blending Mode of the Layer palette. For MS identification, protein spots were excised and in-gel trypsin digestion was performed as described elsewhere [46]. An aliquot (4.5 μL) of the resulting peptide mixture was separated by C18 (75 μm i.d. × 100 mm) (Waters, Milford, MA, USA) RP-HPLC coupled with nanoelectrospray MS/MS on a Q TOF Ultima mass spectrometer (Waters, Milford, MA, USA) at a flow rate of 600 nL/min. The gradient was 0–80% acetonitrile in 0.1% formic acid over 22 min. The instrument was operated in the ‘top three’ mode, in which one MS spectrum is acquired followed by MS/MS of the top three most intense peaks detected. Full dynamic exclusion was used to enhance dynamic range–one spectrum before exclusion for 120 s. The resulting fragment spectra were searched using MASCOT search engine (Matrix Science, UK) against the UniProt database (v. 51.6) restricted to the taxa Serpentes with a parent and fragment tolerance of 0.6 Da. Iodoacetamide derivative of cysteine and oxidation of methionine were specified in MASCOT as variable modifications. Peptide identifications were accepted if they exceeded specific database search engine thresholds (p< 0.05). Protein identifications were accepted if they contained at least 1 identified peptide.

### Detection of proteins similar to batroxobin in venom samples

Batroxobin is a thrombin-like enzyme (SVSP) purified and cloned from *Bothrops atrox* venom [47,48], which shows 65–96% identity to other thrombin-like enzymes purified from *Bothrops* (*sensu lato*) venoms. To test whether batroxobin or similar proteins were expressed in venom from hybrids, Western blotting was used. Briefly, recombinant batroxobin (1 μg/μL, Prospec, Israel) expressed in *Pichia pastoris* was used to immunize one rabbit and obtain polyclonal antibodies. In brief, rabbits were immunized, as described elsewhere [49], by administrating four consecutive boosters of 10 μg recombinant batroxobin in Marcol-Montanide adjuvant; this rabbit serum was used in Western blotting assays. Pooled venom samples (15 μg) from *B*. *atrox* (positive control, from Laboratory of Herpetology, Instituto Butantan), the mother, the father, young hybrids, adult hybrids and individual venom samples from hybrid #15 were electrophoresed under non-reducing conditions in 12% SDS-PAGE gels [43] and transferred onto nitrocellulose membranes (0.22 μm pore size) at 15 V during 2 h, using a semi-dry transfer blotting system (BioRad). Membranes were blocked with skim milk, incubated with anti-batroxobin antibodies (1/2500) and subsequently with 1/10000 peroxidase-conjugated anti-rabbit IgG (Sigma, A0545). The reaction was developed with 3,3-diaminobenzidine plus 2.5 mM CoCl_2_ in imidazole buffer, as previously described [50]. The determination of apparent molecular masses was carried out using the software TotalLab TL100 v2006 (Phoretix, USA).

## Results

### Biological features of hybrids

Most F1 hybrids survived, but three of 15 individuals died over time. As shown in Fig 2, their pattern of coloration was a mixture between the patterns of the father and the mother. In regard to reproduction data from F1, in September 2010, Hy# 11 (female) mated with Hy# 03 (male), and an offspring (F2) of 2 animals was born (also shown in Fig 2) on April 13, 2011, but they survived for only ca. 6 months. In addition, Hy# 05 was bred to Hy# 08, but no offspring was born. In 2013, a female F2 was born from the mating between Hy# 02 and Hy #03. On the other hand, in 2012, Hy# 06 gave birth to 4 males and 1 female after breeding with a male *Bothrops paoloensis*, and a female *B*. *neuwiedi* delivered 4 females and 2 males after copulation with Hy# 08. No venom samples were extracted from F2 hybrids.

**Fig 3:**
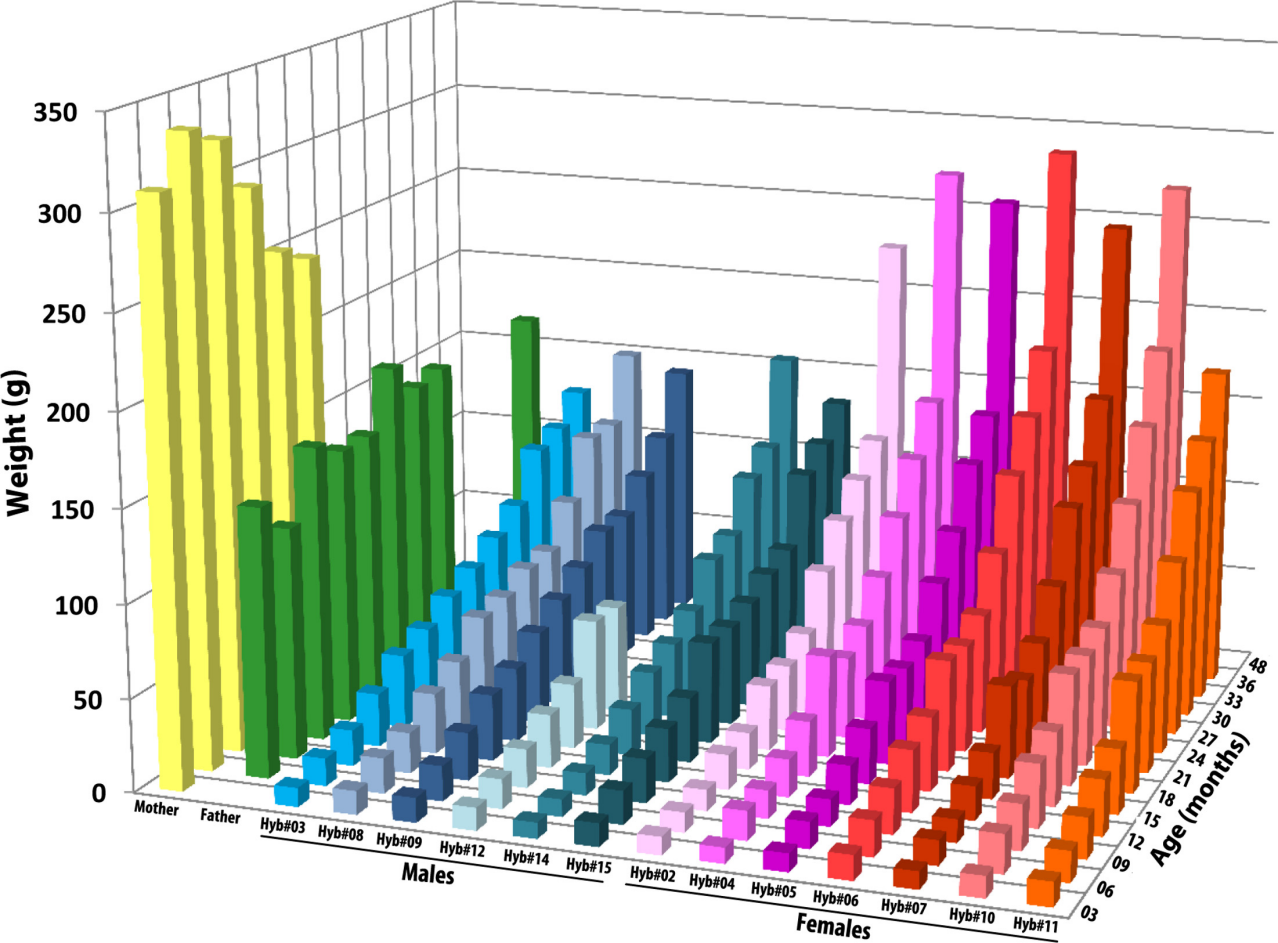
Growth of F1 hybrids determined by weight gain over time. The weights for the father and mother are also shown in the figure for comparison.

Hybrid’s growth, represented by their increase in weight over time, can be observed in Fig 3. Nearly 3 years after birth, female hybrids initiated to become heavier than males, and their weight almost doubled at 4 years old.

### Biological activities of venoms

Due to low amounts of venom extracted from snakes, particularly when hybrids were less than 6 months old, not all analyses could be performed in venom samples. Thus, at 3 months, venom samples from Hy# 03 and Hy# 08 were not tested for amidolytic and collagenolytic activities, and Hy# 03, Hy# 06, Hy# 08, and Hy#11 for coagulant activity. At 6 months, samples from Hy# 08 and Hy# 10 could not be examined for coagulant activity.

**Fig 4:**
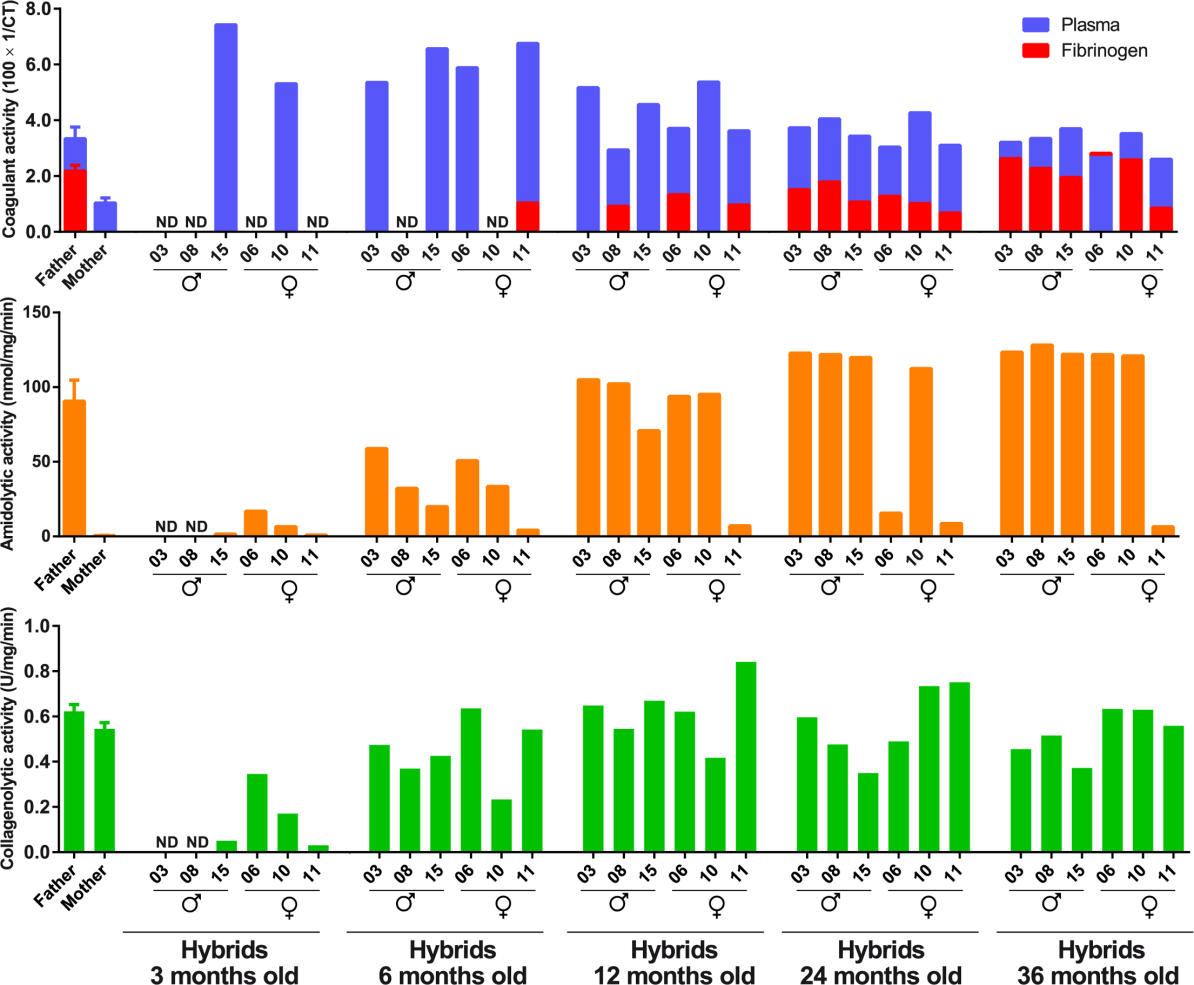
Enzymatic activities of venom samples from the mother (*B*. *erythromelas*), the father (*B*. *neuwiedi*) and hybrid progeny over time. Venom samples were tested for coagulant activity on plasma (blue bars) and fibrinogen (red bars), amidolytic activity (orange bars) and collagenolytic activity (green bars). Data from the mother and father are expressed as mean ± standard error of mean of individual results obtained from venom samples extracted over the period of hybrid growth.

As shown in Fig 4, venom activities varied over the period of hybrid growth, and individual differences were also noticed.

#### Coagulant activity

Typical *B*. *erythromelas* snake venom exhibits a higher coagulant activity than *B*. *neuwiedi*, due to presence of high levels of factor X and prothrombin activators, but it lacks thrombin-like activity [33,38,51]. However, the coagulant activity of the venom from the mother *B*. *erythromelas* (MCD > 200 μg/mL) was idiosyncratically lower than that of the father (*B*. *neuwiedi*), while thrombin-like activity was absent as in other *B*. *erythromelas* venoms. A venom pool from other *B*. *erythromelas* specimens, supplied by the Laboratory of Herpetology (Butantan Institute), displayed a MCD of 5.5 μg/mL, which is close to the value reported elsewhere (4.5 μg/mL) [38], demonstrating that the biological mother did exhibit a extremely low coagulant activity on plasma.

As depicted in Fig 4, the ontogenetic variation in the plasma coagulant activity of venom samples from hybrids was similar to that of other *Bothrops* snakes [38,42], i.e., higher in younger snakes than in adult ones. This activity on venom samples from 3- or 6-month-old hybrids was the most elevated, and gradually decreased over snake growth, reaching a plateau at 24 months and individual values similar to those of the father.

To analyze the thrombin-like activity of venom samples, bovine fibrinogen was used as substrate. Hybrid venoms did not coagulate fibrinogen when they were 3 and 6 months old, similarly to *B*. *erythromelas* venom, except Hy# 11, that showed a low thrombin-like activity at 6 months old. Progressively, thrombin-like enzymes initiated to be detected at 12 months old, and all analyzed hybrids displayed it at 24 and 36 months of age. At the later ages, thrombin-like activity was comparable to that of the father. Among hybrids, Hy# 11 was an exception, once it continued to express low coagulant activity on fibrinogen over its growth.

#### Amidolytic activity

The most striking individual and ontogenetic differences in hybrid venoms were noticed for the amidolytic activity. The venom samples from the father showed a remarkable higher amidolytic activity in comparison with that of the mother. As previously reported [38], both newborn and adult *B*. *erythromelas* venoms show minute amidolytic activity. In hybrid venom samples, this activity was low when hybrids were 3 months old, and progressively increased over time (Fig 4). The venom from Hy# 11, differently from those from other hybrids, showed reduced amidolytic activity over time, which was nonetheless higher than that showed by the mother venom.

#### Collagenolytic activity

Collagenolytic activity is a typical feature of snake venom metalloproteinases (SVMPs) from the family Viperidae. As show in Fig 4, the collagenolytic activities of both the mother and the father venoms were alike. However, like *B*. *jararaca* venom [42], 3-month-old hybrid venoms exhibited lower collagenolytic activity, which increased thereafter. The collagenolytic activity of venoms from 1-year-old hybrids was already similar to that of the parents.

### One dimensional gel electrophoresis

**Fig 5:**
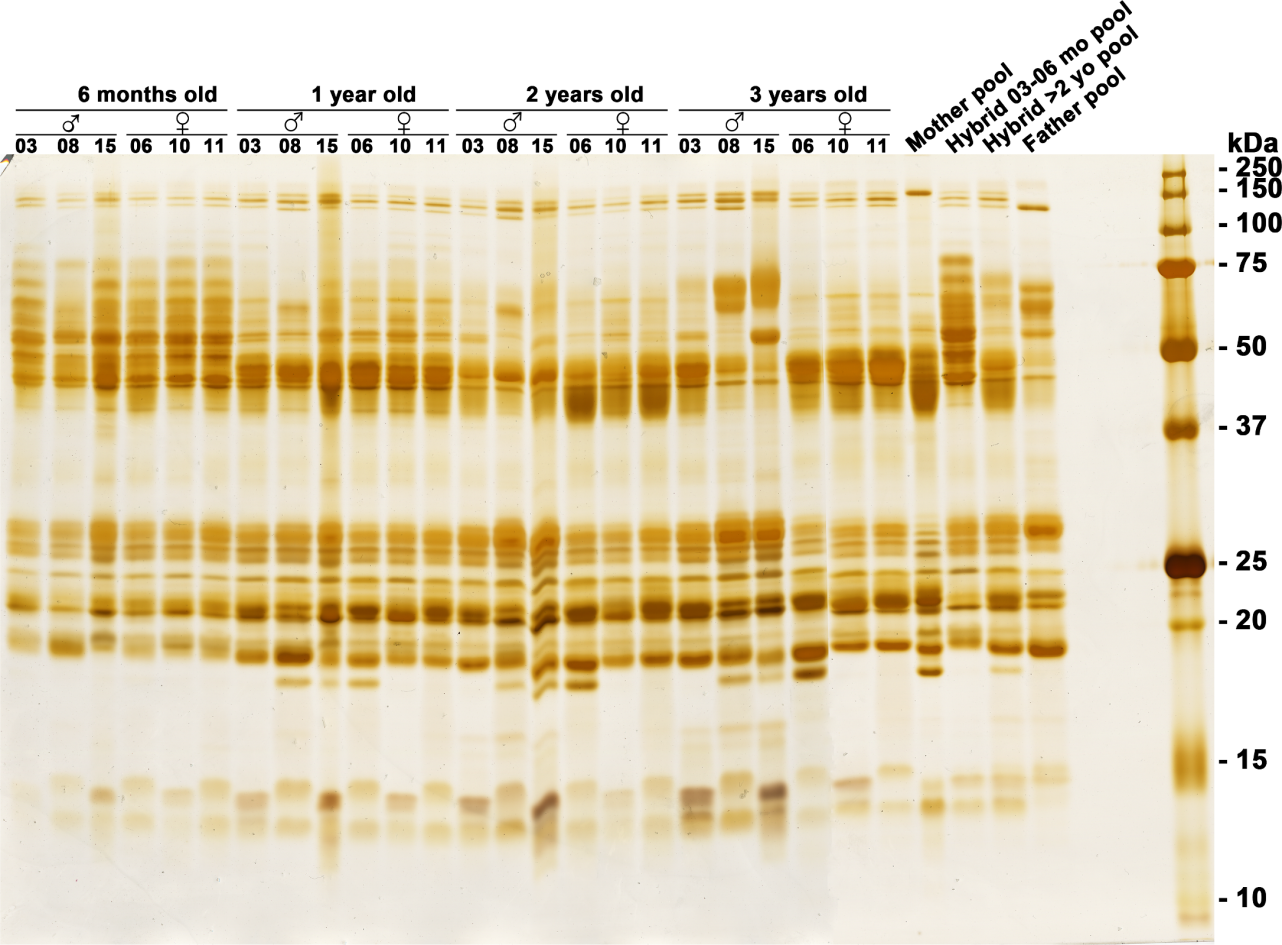
Comparative electrophoretic profile of non-reduced venom samples (20 μg/lane) run in 12% SDS-PAGE gels from individual hybrid snakes over their development. For comparison, the venom pools from the mother, father and hybrids at 3 and 6 months old (mo), and at >2 years old (yo) are also shown. Molecular mass markers are shown on the right. Gels were silver stained.

In general, the electrophoretic profile of venom proteins from 6-month-old hybrids analyzed under non-reducing conditions showed a large quantity of bands with molecular mass between 55 and 125 kDa, which tended to diminish after 1 year of age (Fig 5). However, this pattern was not identical to all hybrids, once the venom from Hy# 08 and Hy# 15 continued to show bands within this region at 1 and 2 years of age, and particularly when they were 3 years old. Moreover, the electrophoretic profile of pooled venom from hybrids at 3 and 6 months showed a similar pattern than those of individual venoms. The mother venom pool showed less stained bands of 55 kDa and above than the father venom. On the contrary, the bands with molecular masses lower than 30 kDa tended to increase in intensity as hybrids grew. Sexual dimorphism among hybrids was noticed particularly when hybrids were 3 years old, especially in the range of bands between 45 and 55 kDa, and 23 and 30 kDa, where proteins from female hybrid venoms showed bands more and less intensely stained, respectively.

Although the pattern of proteolytic and coagulant activity among hybrids of the same age was quite different, the profile of most protein bands in one-dimensional gels was quite similar in hybrids at the same age. Thus, we tested by two-dimensional electrophoresis whether similar patterns of spots would also be noticed for the venoms from selected 3-month-old and 2-year-old hybrids.

### Two-dimensional gel electrophoresis

**Fig 6:**
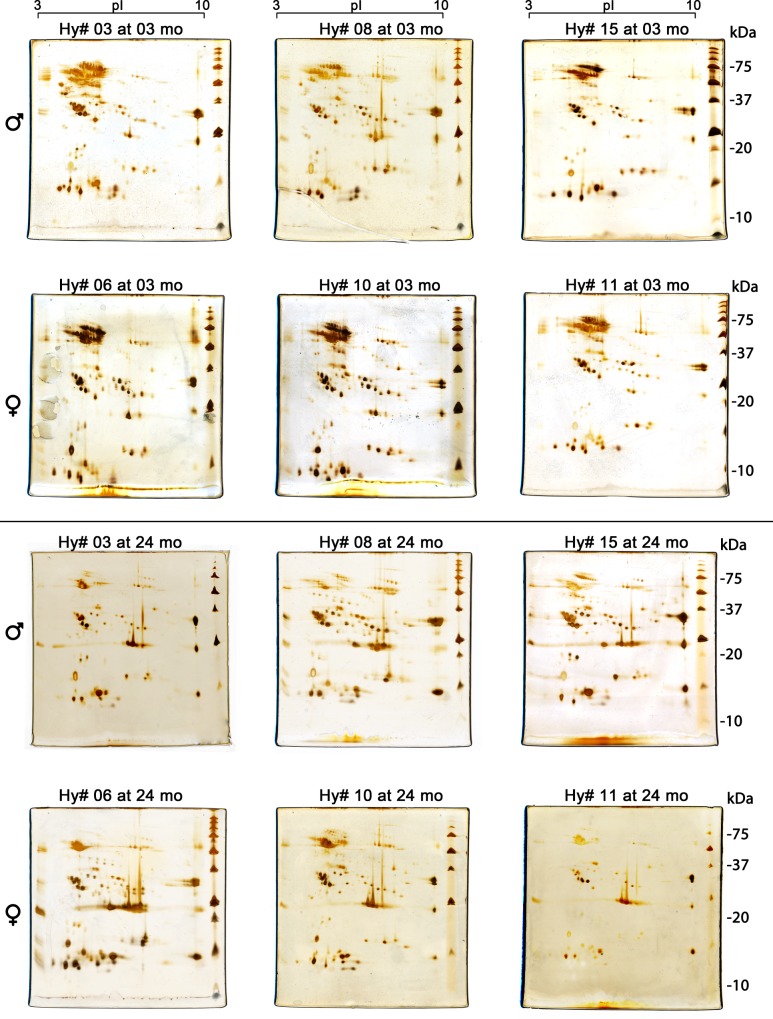
Comparative distribution of protein spots in two-dimensional gel electrophoresis of individual male (Hy# 03, Hy# 08 and Hy# 15) and female (Hy# 06, Hy# 10 and Hy# 11) hybrids at 3 and 24 months old (mo). The images of the biological mother (*B*. *erythromelas*) and father (*B*. *neuwiedi*) pooled venoms are depicted in Fig 7. Gels were run under identical conditions and silver stained. For comparison, boxes were drawn over images of Hy# 03 to facilitate comparison: green dashed boxes: acidic proteins (molecular mass: 50–125 kDa, p*I* 4.0–6.0); blue dashed box: neutral proteins (molecular mass: 23–25 kDa, p*I* 6.5–7.0).

**Fig 7:**
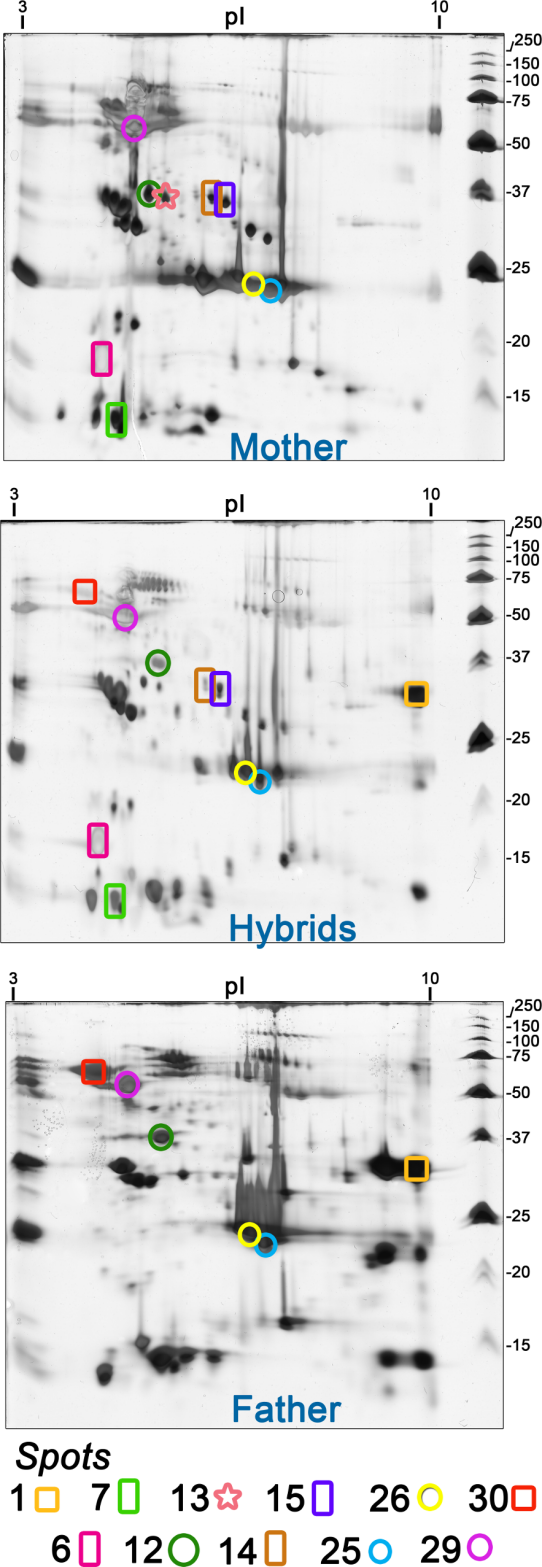
Two-dimensional gel electrophoresis profiles of pooled venoms from the mother (*B*. *erythromelas*), adult hybrids (> 2 years old) and the father (*B*. *neuwiedi*). Gels were run under identical conditions and silver stained. Selected spots were excised from the gels and analyzed by MS.

Protein spots from individual venoms from 3-month-old and 2-year-old hybrids were found in the range of 13–125 kDa, and p*I* from 3 to 10 (Figs 6 and 7). When individual hybrid venoms at 3 months old (Fig 6) were compared to the mother or the father pooled venom, overlapping spots were particularly found in the region of 55–125 kDa (green boxes, Fig 6). Similarly to what was observed by one-dimensional gel electrophoresis, proteins spots in this region decreased in intensity and number at 24 months old, but the amount of lower molecular mass spots of neutral p*I* (23–25 kDa, p*I* 6.5–7.0) (blue boxes) augmented concurrently. Spots in the range of 30–40 kDa, p*I* 4.0–8.0, were similarly distributed among venoms from 3- and 24-months-old hybrids. Although conspicuous differences were evident in spot distribution among hybrids at the same age, when the venoms from the same individual were compared at 3 and 24 months of age, a clear overlay of spot distribution was noticed, suggesting that many proteins were expressed throughout the ontogenetic development of these hybrids and constitute a venom spot signature. However, sexual dimorphism by two-dimensional electrophoresis was not as easily noticeable as by one-dimensional gel electrophoresis.

As depicted in Fig 7, the mother pooled venom showed a remarkable amount of acidic (p*I* 4.1–5.7), high molecular mass spots (70–150 kDa), which were less intense in the pooled father venom. On the other hand, spots between 15 and 32 kDa and acidic p*I* (3.6 to 4.0) were expressed in the father venom, but not in the mother one. By overlapping two-dimensional gel images from individual adult hybrid samples with those of their parents, several common spots were observed. By this comparative image analyses, hybrid venoms seemed to pass from a “erythromelas”-like pattern at 3 months old to a “neuwiedi”-like pattern at 2 years old (Figs 6 and 7). In comparison with the mother venom, the father venom also showed a higher expression of protein spots in the range of 30–40 kDa, which are the characteristic molecular mass range of SVSP.

In order to evaluate the heritability of venom proteins in hybrids, due to the large number of spots among specimens, we pooled the venoms from all adult hybrids (> 2 years old), and further compared their spot distribution with those of pooled venoms from the mother and father (Fig 7). Selected spots were cut from the gels and submitted to MS identification (Table 1). Spots 12 (37 kDa, p*I* 5.3; SVMP and phospholipase A_2_), 25 (23 kDa, p*I* 7.2; SVMP), 26 (22 kDa, p*I* 6.9; SVMP), and 29 (55 kDa, p*I* 4.8; SVMP and L-amino-acid oxidases) occurred in all venom pools. Spots 1 (30 kDa, p*I* 9.8; SVSP and SVMP) and 30 (70 kDa, p*I* 4.1; SVMP) were present only in the father and hybrid venoms. Spots 6 (16 kDa, p*I* 4.2; phospholipase A_2_), 7 (< 15 kDa, p*I* 4.7; C-type lectin-like proteins and SVMP precursors), 14 (36 kDa, p*I* 6.2; SVSP) and 15 (35 kDa, p*I* 6.4; SVSP) were noticed exclusively in the mother and hybrid venoms. Spot 13 (38 kDa, p*I* 5.5; phospholipase A_2_) was found only in the mother.

**Table 1:** Identification of peptides released from *in gel* trypsin digestion of venom protein spots shown in Fig 7 from pooled adult hybrid snakes (H), the mother (M, *B*. *erythromelas*), and the father (F, *B*. *neuwiedi*), by LC-MS/MS and database search.

Spot #	Identified peptides^*^	UniProt entry	Identified Protein,Class^**^
**H1**	LGVHSIK	VSP1_BOTJA	Platelet-aggregating proteinase PA-
	INILDHAVCR		BJ, SVSP
**F1**	INILDHAVCR	DISJ_BOTJA	Venom metalloproteinase jararhagin
	MYELANIVNEIFR		precursor, SVMP
**H6**	CCFVHDCCYGK	PA21B_BOTJA	
	QICECDRVAATCFR		
**M6**	VAATCFR	PA21B_BOTJA	*Bothrops jararaca* PLA_2_ (BJ-PLA_2_),
	VAATCFRDNK		PLA_2_
	CCFVHDCCYGK		
	QICECDRVAATCFR		
**H7**	VFNEPQNWADAEK	ABA4_TRIAB	Alboaggregin-A subunit 4, CTL
	ITVKPDVDYTLNSFAEWR	DISJ_BOTJA	Venom metalloproteinase jararhagin
			precursor, SVMP
**H12**	MYELANIVNEIFR	DISJ_BOTJA	Venom metalloproteinase jararhagin
	ITVKPDVDYTLNSFAEWR		precursor, SVMP
**F12**	SVGIVR	DIST_TRIJE	Zinc metalloproteinase jerdonitin
			precursor, SVMP
**M12**	-.SLVQFETLIMK	PA2_BOTPC	Phospholipase A_2_, PLA_2_
**M13**	-.SLVQFETLIMK	PA2_BOTPC	Phospholipase A_2_, PLA_2_
**H14**	-.VVGGDECNINEHR	VSP1_AGKBI	Ancrod, SVSP
**M14**	WDKDIMLIR	VSP2_BOTJA	Kinin-releasing and fibrinogen-
	FFCLSSK		clotting serine proteinase 2
			precursor, SVSP
**M15**	-.VVGGDECNINEHR	VSP1_AGKBI	Ancrod, SVSP
**H25**	ETVLLNR	DISB_AGKCO	Zinc metalloproteinase acostatin-
	YNSNLNTIR		beta precursor, SVMP
**F25**	ERDLLPR	BAP1_BOTAS	Hemorrhagic metalloproteinase
	YNSNLNTIR		BaP1, SVMP
	SVDVHAPLANLEVWSK		
**M25**	TLTSFGEWR	LEUCA_BOTLC	Leucurolysin-a, SVMP
**H26**	HNPQCILNKP	LEUCA_BOTLC	Leucurolysin-a, SVMP
**F26**	TLTSFGEWR	LEUCA_BOTLC	Leucurolysin-a, SVMP
**M26**	ETDLLNR	HRTD_CROAT	Hemorrhagic metalloproteinase HT-
			D/HT-C precursor, SVMP
**H29**	GMVLPGTK	DISJ_BOTJA	Venom metalloproteinase jararhagin
	NNGDLDKIK		precursor, SVMP
	GNYYGYCR		
	KIPCAPEDVK		
**F29**	NNGDLDKIK	DISJ_BOTJA	Venom metalloproteinase jararhagin
	IPCAPEDVK		precursor, SVMP
	AGGRVK	OXLA_BOTMO	L-amino-acid oxidase precursor,
			LAAO
**M29**	DLIKVEK	LEUCA_BOTLC	Leucurolysin-a, SVMP
	ERDLLPR		
	YNSNLNTIR		
	TLTSFGEWR		
**H30**	YFYCR	HR1B_TRIFL	Hemorrhagic metalloproteinase
			HR1b precursor, SVMP
**F30**	GDEYFYCR	VMSB_TRIST	Zinc metalloproteinase-disintegrin
			stejnihagin-B, SVMP

*According to UniProt database. Underlined residues mean carbamidomethylation of cysteines; (-) means N-terminal peptides (for details regarding protein identification see Materials and Methods);

**Toxin class: CTL, C-type lectin; SVMP, snake venom metalloproteinase; LAAO, L-amino acid oxidase; PLA_2_, phospholipase A_2_; SVSP, snake venom serine proteinase).

### Expression of bands reactive to anti-recombinant batroxobin antibodies

**Fig 8:**
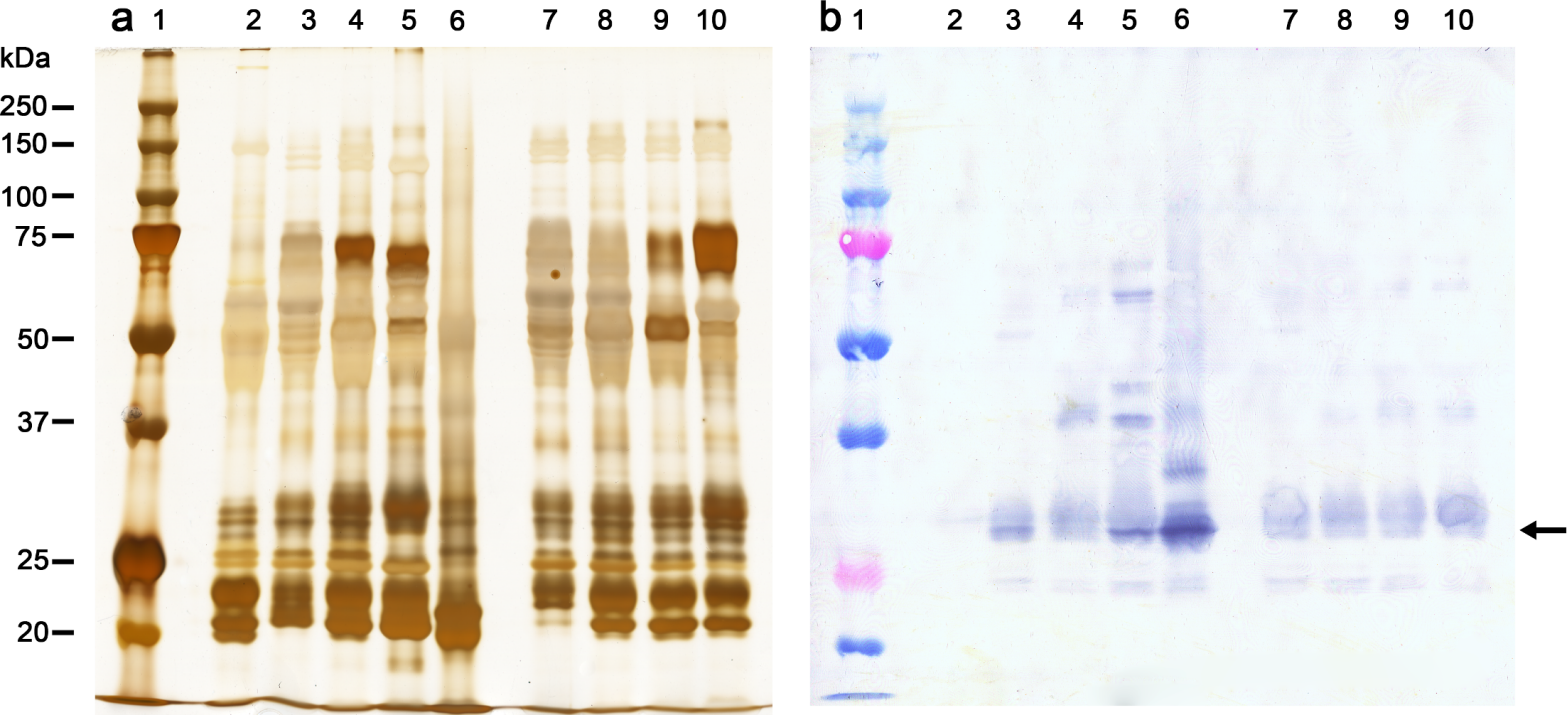
Analyses of venom samples (15 μg, non-reduced conditions) by SDS-PAGE (a) and Western blotting (b). Venom samples: mother pool (*B*. *erythromelas*, lane 2); pooled venoms from hybrids at 3 and 6 months old (lane 3), and adult hybrids (>2 years old) (lane 4); father pool (*B*. *neuwiedi*, lane 5); pooled venom from *B*. *atrox* (lane 6, positive control); and individual venom samples from hybrid # 15 at 6 (lane 7), 12 (lane 8), 24 (lane 9), and 36 (lane 10) months old. SDS-PAGE gels were silver stained [45]. Numbers on the left correspond to the position of molecular mass markers. For Western blotting, nitrocellulose membranes were incubated with anti-recombinant batroxobin (1/2500), and developed as described in Materials and Methods. The arrow indicates the band with molecular mass equivalent to native batroxobin.

Once thrombin-like enzymes (SVSP) were absent in the mother venom, and present in the father venom (*B*. *neuwiedi*), their expression in hybrid venoms was analyzed on ontogenetic development. Most SVSP are single chain proteins, with molecular masses ranging from 26 to 67 kDa, under reducing conditions, depending on their carbohydrate content [52]. Recombinant or native batroxobin from *B*. *atrox* venom has been demonstrated to display molecular masses of 25.5 kDa [53] or 29.1–31.4 kDa [54], respectively. Fig 8A shows the protein profile in SDS-PAGE of venoms studied by Western blotting (Fig 8B). All venom samples showed protein bands in the range of 25.5–31.4 kDa, but when proteins were blotted and probed with the anti-recombinant batroxobin antibody (Fig 8B), the mother venom (lane 2) was noticed to be almost totally devoid of reactive bands, except a faint band at 29 kDa, whereas the father venom (lane 5) showed more densely reactive bands around 27–32 kDa, especially one at 28 kDa, along with other bands of 58, 44, 39 and 24 kDa. The positive control, *B*. *atrox* venom (lane 6), the source of batroxobin, displayed more densely reactive bands than *B*. *neuwiedi* between 27 and 32 kDa, particularly one at 28 kDa, and others with molecular mass higher than 28 kDa. The pooled venom from young (lane 3) and adult (lane 4) hybrids had less densely reactive bands around 27–32 kDa than that of the father venom, but the 28 kDa band was more reactive in the pooled venom of young hybrids. Other faintly stained bands (> 37 kDa) were also noticed in the pooled venom from young (lane 3) and adult (lane 4) hybrids, although the latter showed bands more intensely stained than the former.

Hy# 15 initiated to exhibit thrombin-like activity only at 24 months old (Fig 4). Thus, we analyzed whether protein bands reactive with the anti-batroxobin antibodies appeared during its ontogenetic development. Bands with 27–32 kDa were noticed in all venom samples from Hy# 15, and one reactive band of around 42 kDa appeared from 12 months onwards. However, a strongly stained band with 28 kDa was not observed in the venom of Hy# 15, indicating that enzymes other than batroxobin display thrombin-like activity in this venom.

## Discussion

Few studies have dealt with hybridization and introgressive hybridization in snakes, and most of them have reported external morphological, allozymes, and genetic features of hybrids [16,18,23,26–29,55–60]. Only one study, as far as we know, discussed venom features as an indicator of introgressive hybridization between different snake species [22]. That study investigated immunoreactivity of individual venoms from several populations of *Crotalus viridis viridis* to a polyclonal antibody raised against "Mojave toxin" (a phospholipase A_2_ complex from *Crotalus scutulatus scutulatus*). A strong reactivity was observed only in venoms from snakes obtained from regions where the distribution of both species overlapped, suggesting that the presence of "Mojave toxin" in these populations of *C*. *viridis viridis* was a result of past hybridization with *C*. *scutulatus scutulatus*. Our results from the mating of *B*. *erythromelas* and *B*. *neuwiedi* showed that these species have not experienced complete reproductive isolation yet, and that the hybrid litter did not seem to show reduced genetic or environmental fitness in captivity. These results indicate that genes can still flow from one species to the other, and that this mechanism can be an important factor for genetic variation in both species. The fact that from 15 hybrid siblings only 3 died over the period of time analyzed, and even the occurrence of mating between F1 hybrids, giving rise to F2, favor the hypothesis that hybridization seems possible between these two species in zones where they naturally coexist. However, the higher survival of the progenies from the breeding between F1 hybrids and *B*. *pauloensis* or *B*. neuwiedi, in comparison with those from the breeding among F1 hybrids *inter se*, suggests that the latter may provide maladaptive traits to their offspring. It is plausible to suggest that the breeding between F1 hybrids and their parental species may favor the incorporation of alien genes, and that these hybrids have higher Darwinian fitness. Our results indicate that introgressive hybridization certainly contributes to snake diversity, and, consequently, to venom complexity.

At least for this litter, there was ontogenetic shift for collagenolytic, amidolytic and coagulant activities over hybrid development, and this shift seemed to follow those described for other *Bothrops* venoms [38,42,61–69]. As reported for *B*. *jararaca* venom [61,63], the change in the phenotype of hybrid venom seems to be due to the gradual increase in PI-SVMP and concomitant decrease in P-III SVMP. In the case of hybrids, the synthesis of thrombin-like enzymes (SVSP) in venom glands is profoundly augmented towards adulthood, in contrast with the uniformity in activity reported during the growth of *B*. *erythromelas*, *B*. *neuwiedi*, *B*. *jararaca* and *B*. *jararacussu* [38,42].

SVSP are found in Viperidae, Crotalidae, Elapidae and Colubridae snakes [52], and have been reported to disturb diverse steps of hemostasis, e.g. blood coagulation, fibrinolysis, and platelet function. Among SVSP, thrombin-like enzymes release fibrinopeptide A and/or B from fibrinogen, promoting thereby its clotting [70]. SVSP are also responsible for the venom amidolytic activity on BAPNA, a synthetic substrate for SVSP [52]. *Bothrops neuwiedi* venom exhibits thrombin-like activity [33,38] and amidolytic activity on BAPNA, whilst *B*. *erythromelas* venom lacks thrombin-like activity and amidolytic activity [33,34,38]. Geographic variation in the coagulant activity of *B*. *erythromelas* venom has been reported, so that some specimens collected from Bahia State, where the mother from hybrids came from, showed lower venom coagulant activity on plasma and/or fibrinogen than venoms from specimens collected elsewhere [35,36]. Interestingly, thrombin-like activity was not exhibited by young hybrids, but it was expressed when they approached adulthood. Both adult and neonate *B*. *neuwiedi* express thrombin-like activity, whereas both neonate and adult venoms from *B*. *erythromelas* do not [38]. Hence, we initially suspected thereby that the lack of active genes encoding thrombin-like serine proteinases could be the reason why some *B*. *erythromelas* specimens did not exhibit thrombin-like activity. However, our findings from hybrid venoms suggest that the expression of thrombin-like activity is more complex than the mere presence/absence of active genes. Intact genes encoding enzymes with thrombin-like activity were present in hybrids, since this enzymatic activity initiated to be expressed when they reached adulthood. At the moment, it is difficult to reason how these genes are inherited and controlled in hybrids, or whether active genes were inherited exclusively from the father or from both parents. Although peptide sequences similar to those of thrombin-like enzymes have been identified in *B*. *erythromelas* venom [71] and herein (spot 14), the Western blotting analysis did not reveal reactive bands to the anti-batroxobin antibody in *B*. *erythromelas* venom. However, only faintly reactive bands were noticed in hybrids over their growth, regardless if they were showing or not thrombin-like activity. On the other hand, intensely reactive bands were present in venoms from *B*. *neuwiedi*, *B*. *atrox and B*. *jararaca* venoms (Cunha, B.H.L. and Santoro, M.L., personal observation). Probably these antibodies cross-react with other SVSP, which may demonstrate other biological activities. Nonetheless, since active thrombin-like-enzyme genes seem to be regulated over the period of hybrid growth, it is plausible to speculate that they are under transcriptional, translational or post-translational control. In fact, ontogenetic shift in *Crotalus simus simus* venom has been reported to be regulated by microRNA [72].

Compared to the mother venom, most hybrid venoms in the first six months of life showed a similar lack of coagulant activity on fibrinogen and amidolytic activity on BAPNA, which was expressed thereafter, achieving values similar to those of the father. Such observations generally apply to most hybrid venoms studied, but Hy# 11 was an exception, once it continued to express low coagulant activity on fibrinogen and low amidolytic activity on BAPNA over its growth. Individual variation in the biological activity and protein composition of snake venoms is not a feature exclusive of these hybrids, but it has been noticed in siblings from the same litter of other snake species reared in captivity under controlled conditions [73–75].

Based on MS analyses from spots 29 and 30 and their localization on the gel, spots in green boxes most certainly represent PIII-SVMP in hybrid venoms. Therefore, these proteins are more enriched in venoms from 3-month-old hybrids and the mother than that from the father. The presence of PIII-SVMP in high abundance in adult *B*. *erythromelas* venom, observed here in the mother venom and elsewhere [71], shows that this species shows a pedomorphic trend similar to that reported for *B*. atrox venom found in Brazil, Ecuador and Peru [64,76]. However, the decreased amount of PIII-SVMP in adult hybrid venoms suggests that this trait is dominantly inherited from the father alleles. In adult hybrid venoms, there was an increase in the staining intensity of spots of 20–25 kDa (spots 25 and 26), which were identified as containing PI- or PII-SVMP, similar to that reported for *B*. *jararaca* venom [42,63].

The findings reported herein partially disagree with the hypothesis that the features of hybrid venoms are a combination of those of genitors [20]. In this report, the venom of two putative natural hybrids between *Protobothrops elegans* and *Protobothrops flavoviridis* were analyzed by proteomic techniques, and compared to the venom of parenteral hybrid snakes. Hybrid venoms expressed all phospholipases A_2_ from *P*. *elegans* and *P*. *flavoviridis*, whilst most of, but not all, PII-SVMP, SVSP and C-type lectins present in the latter venoms were found in hybrid venoms. Once only two natural hybrid venoms were studied, the genitors were not known, and no information was available about the age of hybrids at the moment of venom extraction, it is difficult to correlate our findings with those ones. Our findings do evidence that some traits are inherited from the father, others from the mother, and both seem to be under direct genetic control over snake growth.

In conclusion, we demonstrate that venoms from hybrid *Bothrops* snakes undergo a shift in their features over animal growth, as that reported for other *Bothrops* venoms. Our data indicating an ontogenetic control on the expression of thrombin-like enzymes underscore an additional factor for the complexity of snake venoms, be it in natural or hybrid snakes. Moreover, our data suggest that introgressive hybridization in *Bothrops* snakes is certainly an event that accounted and still accounts for the diversification of closely-related snakes, and thereby of their venoms.
